# Parallelogram based approach for *in vivo* dose estimation of genotoxic metabolites in humans with relevance to reduction of animal experiments

**DOI:** 10.1038/s41598-017-17692-5

**Published:** 2017-12-14

**Authors:** Hitesh V. Motwani, Cecilia Frostne, Margareta Törnqvist

**Affiliations:** Department of Environmental Science and Analytical Chemistry, Stockholm University, SE-10691 Stockholm, Sweden

## Abstract

When employing metabolism studies of genotoxic compounds/metabolites and cancer tests for risk estimation, low exposure doses in humans are roughly extrapolated from high exposure doses in animals. An improvement is to measure the *in vivo* dose, i.e. area under concentration-time curve (AUC), of the causative genotoxic agent. In the present work, we propose and evaluate a parallelogram based approach for estimation of the AUC of genotoxic metabolites that incorporates *in vitro* metabolic data and existing knowledge from published *in vivo* data on hemoglobin (Hb) adduct levels, using glycidamide (GA) as a case study compound that is the genotoxic metabolite of acrylamide (AA). The estimated value of AUC of GA per AUC of AA from the parallelogram approach *vs*. that from Hb adduct levels measured *in vivo* were in good agreement; 0.087 *vs*. 0.23 in human and 1.4 *vs*. 0.53 in rat, respectively. The described parallelogram approach is simple, and can be useful to provide an approximate estimation of the AUC of metabolites in humans at low exposure levels for which sensitive methods for analyzing the metabolites are not available, as well as aid in reduction of animal experiments for metabolism studies that are to be used for cancer risk assessment.

## Introduction

Cancer-epidemiological studies that demonstrate association between cancer and exposure is generally regarded as an ultimate proof that the exposure constitutes a cancer risk to human. For assessment of cancer risk from chemicals, toxicological data from animals, or if possibly available from humans, are needed since epidemiological data useful for risk estimation are rarely available. A concept of *in vivo* dose of the causative genotoxic agent, together with its relative genotoxic potency, has been proposed to be used in a model for cancer risk estimation of genotoxic chemicals^1,2^. The *in vivo* dose is defined as the time integral of the concentration of the compound, or as the area under concentration (C) - time (t) curve (AUC), expressed in M × h, which reflects the ADME (absorption, distribution, metabolism and excretion) parameters. The AUC in blood of an electrophilic, genotoxic, chemical in an exposed individual can be inferred from the measured level of formed reaction products (referred to as adducts) to hemoglobin (Hb). Hb adduct levels have been quantified as a measure of the internal dose, i.e. AUC, of several electrophilic genotoxic compounds in exposed animals or in humans with occupational or other exposure [cf. review by Törnqvist *et al*.^3^.

**Figure 1:**
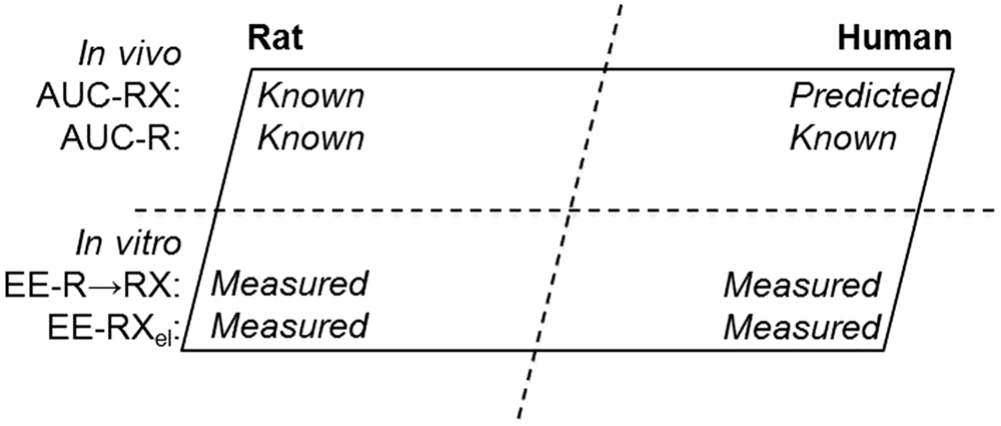
Illustration of the parallelogram approach for inter-species extrapolation of AUC of a reactive metabolite in human using *in vitro* metabolic data. For a metabolic transformation of R to RX, AUC-RX and AUC-R refer to the *in vivo* dose of RX and R, respectively, and EE-R → RX and EE-RX_el_ refer to the *in vitro* enzyme efficiency for metabolic transformation of R to RX and elimination of RX, respectively.

There is an increasing demand for risk assessment of an escalating number of chemicals produced, or formed as by-products, prompting a paradigm shift from whole animal testing. Simultaneously, there is a need by the regulatory organizations for new prediction models based on *in vitro* methods emphasizing the 3 R (replacement, reduction and refinement) principles with regard to animal experiments. This is put in perspective, for instance, by the US Environmental Protection Agency (EPA) driven guideline to reduce the need of animals for toxicity testing^4^, and the recommendation by European Food Safety Authority (EFSA) on applying 3 R principles in risk assessment approaches^5^. Within these frames we have earlier proposed a parallelogram approach for inter-species extrapolation of AUC of electrophilic genotoxic metabolites to be used in risk assessment procedures^6^. This approach is based on *in vitro* methods, integrated with knowledge from available *in vivo* data (Fig. 1). The basic concept is that AUC of a genotoxic metabolite in human, which is challenging to measure, can be obtained by extrapolation from measured protein adduct levels of that metabolite formed in mice/rats and through comparison with *in vitro* formation/elimination of the metabolite in human and the studied rodent^6^.

Using the parallelogram approach, for 1,3-butadiene (BD) as the first model compound, the *in vivo* dose of BD’s most potent genotoxic metabolite, 1,2:3,4-diepoxbutane (DEB), was predicted in human. The inter-species extrapolation was performed using *in vitro* metabolic data on enzyme kinetics and published Hb adduct data^6^. This study was initiated as the AUC of DEB (AUC-DEB), in humans exposed to BD, had not been possible to measure with available methods. The AUC predicted with the parallelogram approach showed a good agreement with the AUC-DEB that finally had been attained when measurement of Hb adduct levels of DEB in humans was achieved^7^.

**Figure 2:**
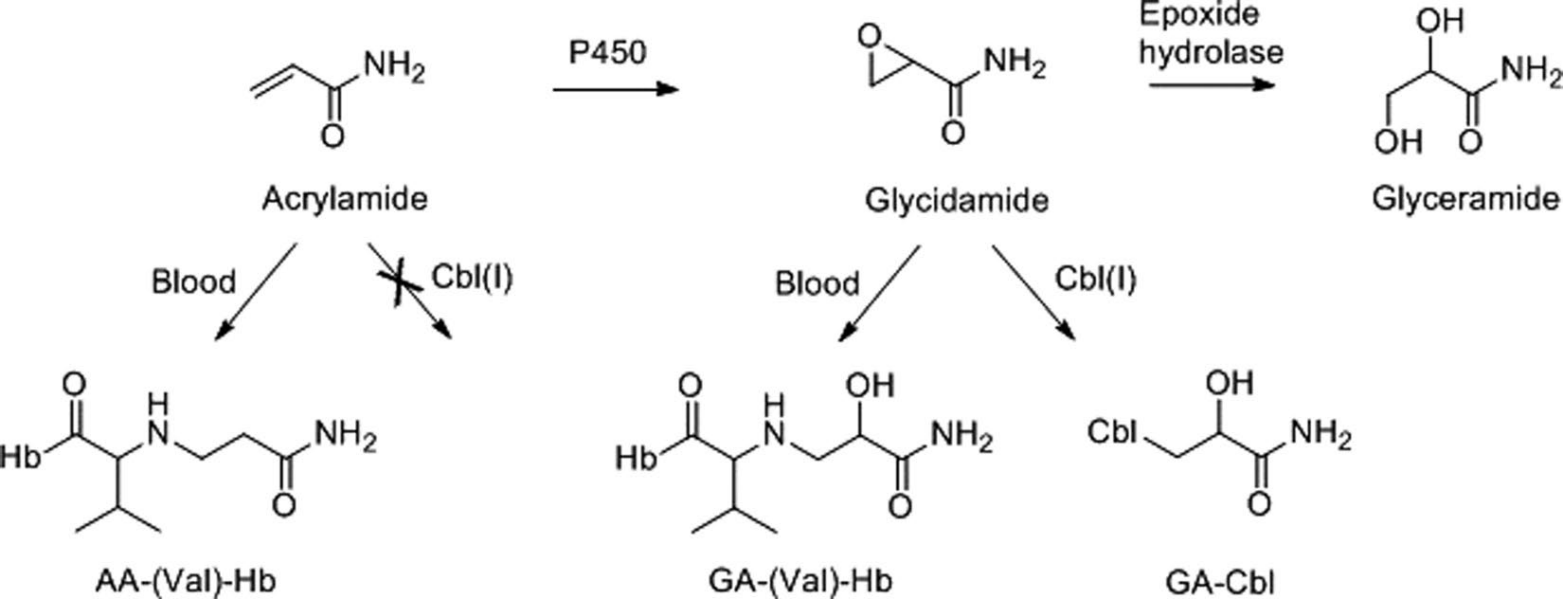
Scheme showing metabolism of acrylamide (AA) and formation of corresponding adducts. AA and glycidamide (GA) can form adducts to hemoglobin at N-terminal valine; represented as AA-(Val)-Hb and GA-(Val)-Hb, respectively. GA can react with cob(I)alamin [Cbl(I)], unlike AA, to form the cobalamin adduct GA-Cbl.

In the present work, the parallelogram approach is explored in a case study on toxicokinetics of glycidamide (GA), a metabolite of acrylamide (AA). The epoxy metabolite of AA, i.e. GA, is formed by oxidative metabolic transformation of AA by cytochrome P450 (P450) 2E1 (Fig. 2)^8–10^. Both AA and GA undergo glutathione conjugation, and GA can be hydrolyzed by epoxide hydrolase. The high-volume industrial chemical AA in its polymeric forms is widely used in waste water treatment, mineral and crude-oil processing, pulp-paper industry, concrete and grouts and thus occurs in occupational exposures^9,11^. There is also exposure to AA through food as the compound is formed in food during preparation at high temperature^12^, simultaneously with formation of other compounds in the Maillard reaction process^13^. AA is shown to be carcinogenic in animal experiments, and is classified as “probably carcinogenic to humans”^14^. More recent studies have shown that both AA and GA are carcinogenic in animal cancer assays^15,16^. GA is considered to be the cancer-risk increasing agent from AA exposure, as the epoxide forms adducts to DNA and is shown to be the genotoxic agent in animal studies with AA exposure^9,15,17,18^. AA has become a toxicologically well-studied compound and data are available on Hb adduct levels for both AA and GA, including AUC of AA (AUC-AA) and AUC of GA (AUC-GA), from rats and humans exposed to AA. The transformation of AA to GA is apparently suitable for testing the parallelogram approach, as it is possible to validate the predicted AUC with available *in vivo* data, and thus evaluate the approach.

A prerequisite to perform the parallelogram based extrapolation to predict AUC of a metabolite in human is to have accurate quantitative *in vitro* metabolism data for formation and disappearance of the concerned metabolite in human and another species (e.g. rat or mouse) (Fig. 1). For accurate measurements of an electrophilic compound we have earlier developed a method where the electrophile is trapped with the reduced form of vitamin B_12_, cob(I)alamin, which results in formation of an alkylcobalamin, a stable derivative form of the electrophilic species^8,19,20^. This so called cobalamin-adduct method was employed in the present study for measuring the metabolic formation of GA from AA, and to follow disappearance of the epoxide in an *in vitro* metabolic system. We herein obtain enzyme kinetic data for the metabolism involving AA and GA in liver S9 fractions of human and rat, and apply the parallelogram approach for estimation of AUC-GA in human based on inter-species comparison of metabolism and *in vivo* dose. AUC-GA obtained from the parallelogram approach and the AUC from earlier measured Hb adducts *in vivo* are compared and are quantitatively evaluated as a basis for validation of the parallelogram approach. This approach is in accordance with the 3R principles to enable application of *in vitro* methods in risk assessment procedures of genotoxic compounds.

## Results

### Quantification of GA

The epoxide GA, either as product from AA metabolism or as substrate, was quantified in the S9 metabolic system using the cobalamin-adduct method. In this method, reduced form of vitamin B_12_, cob(I)alamin (Supplementary information Note 2), is used for trapping the electrophilic metabolite. Trapping by the supernucleophile cob(I)alamin is almost instantaneous and results in the formation of corresponding cobalamin adduct, i.e. GA-Cbl (Fig. 2), which was characterized by LC-MS/MS (Supplementary information Notes 3–4). Earlier, the cobalamin method has been validated for analysis of GA from S9 matrix, where it was shown that the calibration curve was linear over a GA concentration from 200 μM down to 0.01 μM with coefficient of variation in the range 1.68–16.7%^8^.

### *In vitro* metabolism involving AA and GA

Metabolism studies were performed using liver S9 fractions of human (5 pooled male) and rat [male, Sprague-Dawley (SD)], with AA (*n* = 8 × 2) and GA (*n* = 7 × 2) as substrates, using propylene oxide (PO) as an internal standard. Formation of GA, catalyzed by P450, with AA as substrate, as well as elimination of GA was measured under the metabolic conditions. Substrate concentrations for AA and GA and respective incubation time, as given under Methods, were selected based on preliminary experiments to fit Michaelis-Menten kinetics. Representative LC- MRM chromatogram from the S9 metabolism studies showing GA-Cbl from GA and PO-Cbl from the internal standard is shown in Supplementary information Fig. 1. GA-Cbl peak was not detected in absence of NADP when studying P450 oxidation of AA to GA, and in absence of the respective substrates. Chemical reaction of proteins with the electrophilic compounds AA and GA, and nonenzymatic hydrolysis of GA to glyceramide during the incubation, were assumed to have no significant influence on the enzyme kinetic measurements. This assumption is based on control experiments in our earlier work on 1,2-epoxy-3-butene (EB), a metabolite of BD, where the disappearance of the epoxide due to reaction with proteins at similar incubation conditions was shown to be negligible in heat inactivated S9 (and the difference in reactivity between these compounds is not that large)^6^. Moreover, the rate of hydrolysis of GA in an aqueous solution has been determined earlier as 0.016 h^−1^ (half-life 43 h) at 37 °C^21^, indicating no significant nonenzymatic hydrolytic disappearance of GA under the incubation period of 10–15 min.

**Figure 3:**
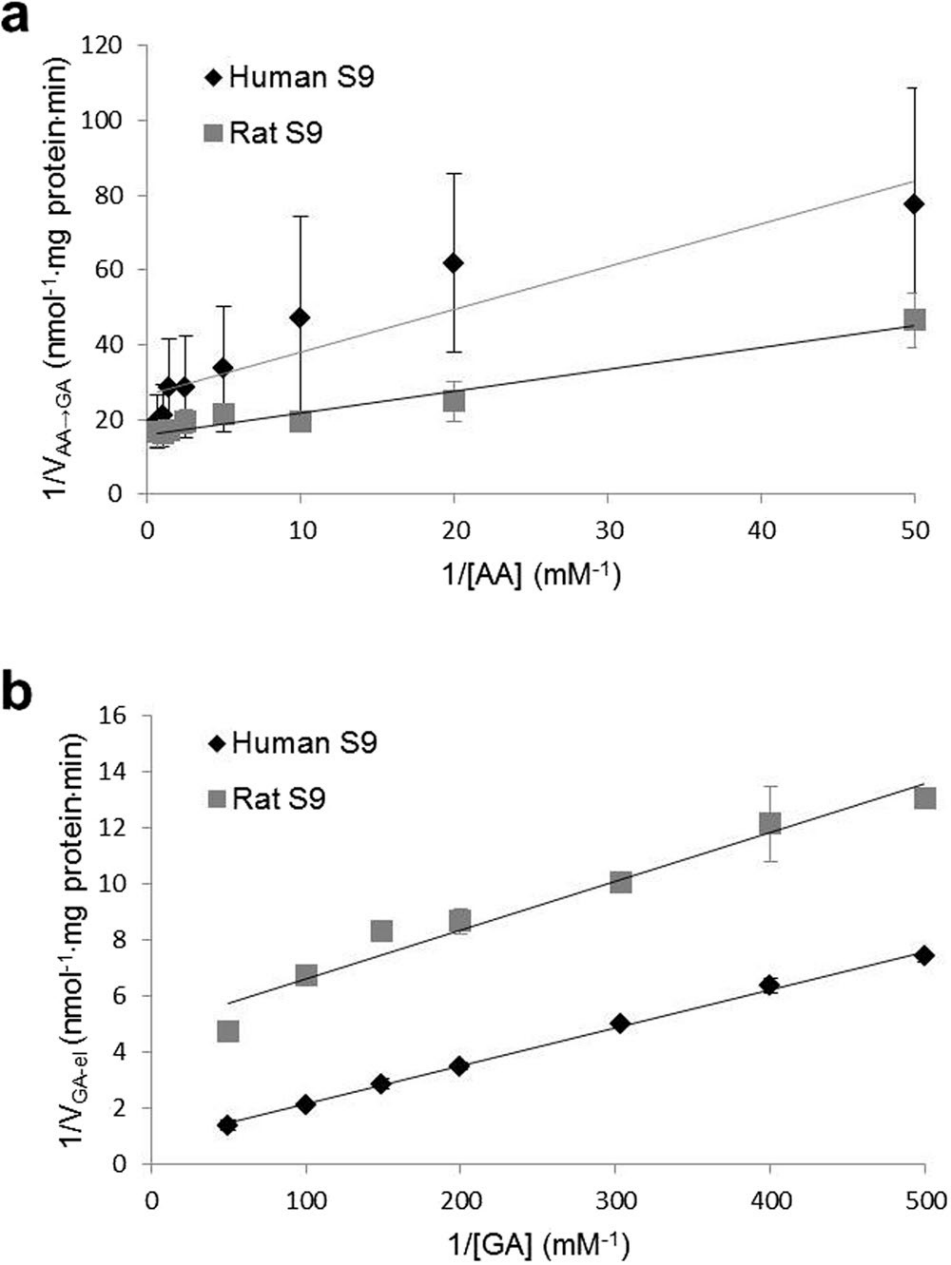
Lineweaver-Burk plots showing regression lines with error bars for metabolic transformation of AA to GA (**a**) and elimination of GA (**b**) using human S9 and rat S9.

Double reciprocal plots using the Lineweaver-Burk equation for the *in vitro* metabolism studies in human and rat are illustrated in Fig. 3a and b, with AA and GA as substrates, respectively. Correlation coefficients (r^2^) of the plots from the two species for AA to GA (AA → GA) were 0.87–0.96 (Fig. 3a) and for GA elimination (GA_el_) were 0.95–0.99 (Fig. 3b). Apparent enzyme kinetic parameters, V_max_ and K_m_, for the metabolic steps AA → GA and GA_el_ were determined by nonlinear fitting using the graphing software Origin. The obtained values are given in Table 1, with respective standard errors. As the parameters V_max_ and K_m_ were determined by the same experimental setup, the enzyme efficiency (EE) for the metabolic reactions is calculated as the ratio of V_max_ to K_m_. Table 1 includes EE values in μL·mg protein^−1^·min^−1^, which represents the second-order rate constant for the corresponding enzymatic reactions and correlates to the intrinsic hepatic clearance that would occur *in vivo*.

**Table 1:** Kinetic constants for metabolic transformation of AA to GA and elimination of GA.

Metabolic pathway	Human S9			Rat S9		
	V_max_ (SE)^c^, nmol·mg protein^−1^·min^−1^	K_m_ (SE), mM	EE^d^	V_max_ (SE), nmol·mg protein^−1^·min^−1^	K_m_ (SE), mM	EE
^a^AA to GA	0.053 (0.007)	0.11 (0.06)	0.50	0.060 (0.004)	0.029 (0.007)	2.1
^b^GA elimination	1.6 (0.7)	0.026 (0.02)	62	0.26 (0.02)	0.0060 (0.001)	43

^a^ *n* = 8 × 2.

^b^ *n* = 7 × 2.

^c^Standard error in parenthesis.

^d^EE = V_max_/K_m_, μL·mg protein^−1^·min^−1^.

Comparing EE between the species, the oxidative formation of GA was ca. 4 times faster in rat than in human, and the rate of elimination of GA was ca. 1.5 times faster in human compared to the rat (Table 1). The EE ratio of AA → GA to that of GA_el_ thereby was ca. 6 times higher in the rat compared to human. Such a comparison of the relative metabolism between species is possible as the metabolic transformations were studied using same conditions for the incubation system (liver S9) and the accurate analysis of GA obtained by the cobalamin method.

Using freshly isolated hepatocytes might provide better resemblance of physiological conditions than that by liver S9, which was used in the present study. However, advantages of the S9 are that it is easily available and simple to use, and an evaluation of the data in Table 1 and earlier studies^6,20^ suggest it to be adequate for the present metabolism studies. There are no earlier studies on *in vitro* enzyme kinetic measurements of GA elimination to our knowledge. The inter-species difference between the rates for AA to GA is in accordance with an earlier study using liver microsomes followed by analysis of GA with an isotope dilution LC-MS/MS method, where the oxidation to GA in rat (male, SD) was ca. 3 times faster than in human (male)^22^. The minor difference between the present study and that by Tareke *et al*.^22^ is probably mainly due to the different metabolic systems and analytical methods.

### *In vivo* dose of AA and of GA from published Hb adduct levels

For the inter-species extrapolation with the parallelogram approach, and for the comparison of results with *in vivo* data, two published exposure studies were used. The mean AUC of AA and of GA given in Table 2 were obtained from a study in humans [number of individuals (n) = 9]^23^ with increased dietary intake of AA, and from a study in rats (n = 6)^24^ with exposure to AA through drinking water, as described under Methods. The best estimate of the metabolism of AA to GA for inter-species extrapolation would be to consider the ratio of AUC-GA to AUC-AA, instead of using, e.g., AUC-GA per exposure dose of AA. This is because AUC-AA corrects for uncertainties in estimated exposure dose, particularly challenging to know accurately from dietary intake, as well as that for individual differences in pharmacokinetics of AA.

**Table 2:** AUC of AA and of GA in human and rat calculated from measured Hb adducts.

AUCs (in nM·h/μg AA per kg bw)	Human	Rat
AUC-AA	212 ± 56	34 ± 3
AUC-GA	49 ± 17	18 ± 1
AUC-GA per AUC-AA	0.23 ± 0.05	0.53 ± 0.07

*Note:* Values given as mean ± standard deviation.

Human data from intake of ca. 11 μg AA/kg bw and day (n = 9), repeated daily dosing during 4 days through food^23^. Rat data from exposure to two different doses, 500 and 2000 μg AA/kg bw and day (n = 3 at each dose), repeated daily dosing during one week through drinking water^24^.

A ratio of AUC-GA to AUC-AA from the published studies according to above was taken for comparison between the species; AUC-GA per AUC-AA in human and rat was 0.23 and 0.53, respectively (Table 2). The AUC per exposure dose of AA in human showed a coefficient of variation of about 27% and 35% for AA and GA, respectively^23^. These relatively small variations could be assumed to be due to uncertainties in the estimates of intake, and individual differences in absorption and metabolism of AA, as well as that in metabolism of GA (cf. refs^25,26^). The coefficient of variation in the AUC of AA and of GA per exposure dose of AA in the rat was lower, about 15% and 7%, respectively^24^. Supplementary information Note 5 shows the range of AUC-AA and AUC-GA, between published data, from oral exposure to AA in humans and rats.

### *In vivo* dose of GA predicted from the parallelogram approach

The parallelogram approach representing inter-species comparison of *in vivo* dose and the use of *in vitro* data is shown in Fig. 1 for the metabolic transformation of R to RX, which is referred to as the metabolism of AA to GA in the present study. This figure can be formulated to Eq. 1 as described in Methods (cf. Eq. 5), where the superscripts H and R represent the corresponding AUC or EE in human and rat, respectively.

1 $$\frac{{{\rm{AUC}}}^{{\rm{H}}}-{\rm{RX}}}{{{\rm{AUC}}}^{{\rm{R}}}-{\rm{RX}}}=\frac{\frac{{{\rm{EE}}}^{{\rm{H}}}-{\rm{R}}\to {\rm{RX}}}{{{\rm{EE}}}^{{\rm{H}}}-{{\rm{RX}}}_{{\rm{el}}}}}{\frac{{{\rm{EE}}}^{{\rm{R}}}-{\rm{R}}\to {\rm{RX}}}{{{\rm{EE}}}^{{\rm{R}}}-{{\rm{RX}}}_{{\rm{el}}}}}\times \frac{{{\rm{AUC}}}^{{\rm{H}}}-{\rm{R}}}{{{\rm{AUC}}}^{{\rm{R}}}-{\rm{R}}}$$

The *in vitro* data on enzyme kinetics from human and rat (Table 1), and the *in vivo* data on AUC of AA and of GA in rat (Table 2) were used to calculate the predictive value of AUC-GA per AUC-AA in human, according to the parallelogram approach (cf. Methods, Eq. 5). This was calculated to 0.087 (Table 3). If instead, for validation purpose, the corresponding calculation was done for rat using the measured human *in vivo* data, together with the *in vitro* metabolic data, AUC-GA per AUC-AA in rat was predicted to 1.4. Incorporating the measured *in vivo* data on AUC-AA from human and rat (Table 2) in Eq. 5, the AUC-GA in human and rat was calculated to 18 and 48 nM·h, respectively, per exposure dose of 1 μg AA per kg body weight (bw) (Table 3). It was observed that the AUC-GA in human and rat predicted by the parallelogram approach and from measured Hb adduct levels, respectively, differed with about a factor of 3 (cf. Tables 2 and 3). This difference is within the variability range of AUC-GA for respective species calculated from different *in vivo* studies^27^.

**Table 3:** AUC-GA in human and rat estimated from the parallelogram approach.

AUCs (in nM·h/μg AA per kg bw)	Human	Rat
AUC-GA per AUC-AA	0.087^a^	1.4
AUC-GA	18^a^	48

^a^The values were calculated as follows.

$$\frac{{{\rm{AUC}}}^{{\rm{H}}}-{\rm{GA}}}{{{\rm{AUC}}}^{{\rm{H}}}-{\rm{AA}}}=\frac{\frac{{{\rm{EE}}}^{{\rm{H}}}-{\rm{AA}}\to {\rm{GA}}}{{{\rm{EE}}}^{{\rm{H}}}-{{\rm{GA}}}_{{\rm{el}}}}}{\frac{{{\rm{EE}}}^{{\rm{R}}}-{\rm{AA}}\to {\rm{GA}}}{{{\rm{EE}}}^{{\rm{R}}}-{{\rm{GA}}}_{{\rm{el}}}}}\times \frac{{{\rm{AUC}}}^{{\rm{R}}}-{\rm{GA}}}{{{\rm{AUC}}}^{{\rm{R}}}-{\rm{AA}}}.$$

$$\frac{{{\rm{AUC}}}^{{\rm{H}}}-{\rm{GA}}}{{{\rm{AUC}}}^{{\rm{H}}}-{\rm{AA}}}=\frac{\frac{0.50}{62}}{\frac{2.1}{43}}\times \frac{18}{34}=\mathrm{0.087.}$$

$${{\rm{AUC}}}^{{\rm{H}}}-{\rm{GA}}=0.087\times {{\rm{AUC}}}^{{\rm{H}}}-{\rm{AA}}{\rm{.}}$$

$${{\rm{AUC}}}^{{\rm{H}}}-{\rm{GA}}=0.087\times 212\,{\rm{nM}}\,\cdot \,{\rm{h}}/\mu g\,\,\mathrm{AA}\,\,\mathrm{per}\,\,{\rm{kg}}\,{\rm{bw}}$$

$${{\rm{AUC}}}^{{\rm{H}}}-{\rm{GA}}=18\,{\rm{nM}}\,\cdot \,{\rm{h}}/\mu g\,{\rm{AA}}\,{\rm{per}}\,{\rm{kg}}\,{\rm{bw}}{\rm{.}}$$

The *in vivo* data on human and rat used in the parallelogram approach herein were selected from studies by Vikström *et al*.^23^ and Törnqvist *et al*.^24^, respectively. This was because these studies applied the same method for measurement of the Hb adduct levels to N-termini, used the same calibration standards for measured adducts, and were performed at the same laboratory (our laboratory), which altogether reduce uncertainties in inter-species comparison of the AUCs. Further, both the selected studies have relevance for the *in vivo* dose extrapolation in cancer risk estimation of AA from intake via food due to the oral exposure route, dose rates, and exposure duration.

### Uncertainties involved in the parallelogram approach

For the prediction of AUC-GA in human (or rat) from the parallelogram approach, we have integrated data from *in vitro* metabolic studies and earlier *in vivo* studies. One concern is the relevance of exposure levels used in the *in vivo* studies compared to the concentrations in the *in vitro* studies. Approximately, the *in vivo* levels of AA in the studies concerning humans and rats are estimated to be about 100 nM and 25 µM, respectively, whereas the initial substrate concentrations in the *in vitro* studies for AA and GA were 20–1500 μM and 2–50 μM, respectively. Even though the *in vitro* concentrations used are higher than those corresponding to *in vivo*, they are well below than what could cause saturation of the metabolism. Moreover, the *in vivo* data from the human study used in the present comparison (Table 2) is in good agreement with the data published by Fennell *et al*.^28^ who used 10–100 times higher exposure levels of AA in acute oral exposure. Also, the ratio between the *in vivo* AUCs (GA/AA, Table 2) is in good agreement with a human study with lower dietary intake, close to the average background exposure to AA from food^23^ (cf. Supporting Information Note 5).

Another issue is intra- and inter-individual variations in metabolism in humans. This could be due to polymorphism of genes coding for the specific metabolizing enzymes. It is also known that other components in diet, like garlic and ethanol, could have an impact on the metabolism^29,30^. Further, it is known that food prepared at high temperature contains a range of compounds^13^, either inherently reactive or via metabolism, but their effect, e.g., interaction with metabolism of AA and GA is not well known. Vikström *et al*. (2012) observed rather large inter-individual variation (up to a factor of 10) in the Hb adduct levels ratio of GA-to-AA, in a cohort of 68 persons with normal exposure to AA via food^31^. In the human study used here (Table 2) with AA intake from food, there is simultaneous exposure to other compounds in food. Furthermore, the human *in vivo* data as well as the *in vitro* data used for the parallelogram calculations represent mean values from relatively small groups.

There is relatively good agreement between *in vivo* data for rats on AUC of AA and of GA from different AA exposure studies obtained by various methods, as reviewed by DeWoskin *et al*.^27^. For the male rat, the range of AUC-AA and AUC-GA was 18–80 nM·h and 13–52 nM·h, respectively, per μg AA per kg bw^27^. The variations between studies were reduced when comparing AUC-GA per AUC-AA, thus supporting the use of the AUC ratio of GA-to-AA (cf. Supporting information Note 5).

In the *in vitro* enzyme kinetics study performed, SD strain rat (male) was used, whereas Fischer 344 (F344) strain (male) was used in the *in vivo* study for AUC calculation^24^. However, in a study using the SD strain (male), at a similar exposure level of AA, with subchronic exposure through intraperitoneal injections, the AUCs was calculated to 28.2 and 16.3 nM·h per μg AA per kg bw, for AA and GA, respectively, from measured Hb adducts on cysteine site^32^. This shows that the AUCs were in a close range to the data for the F344 rat (presented in Table 2) indicating no major difference in pharmacokinetics between the two strains.

### Validation of the parallelogram approach and its limitations

**Figure 4:**
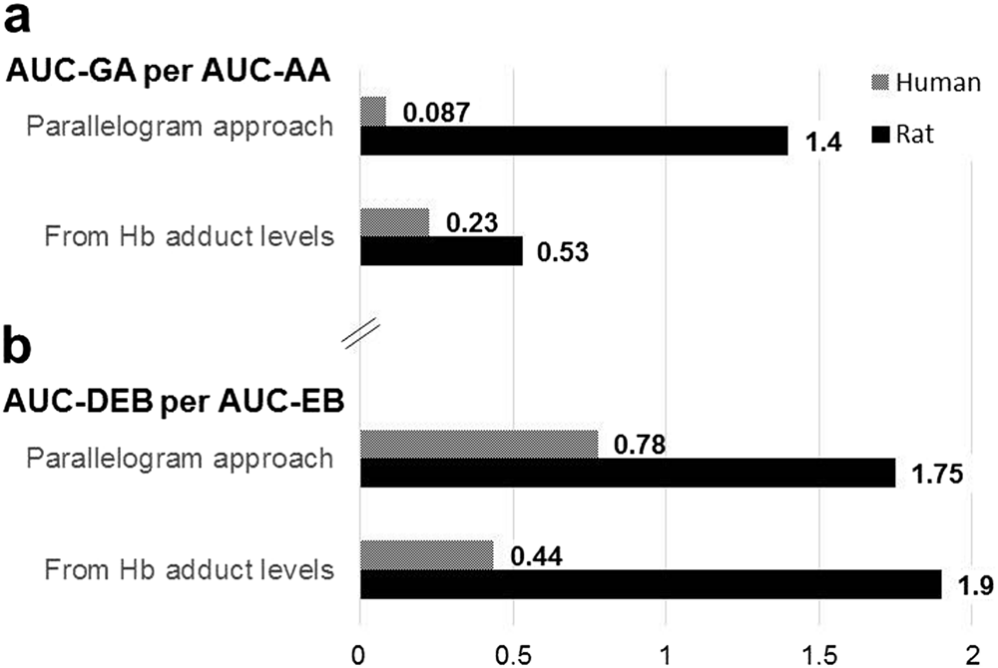
Comparison of AUC of the genotoxic metabolite (per AUC of the precursor) derived from the parallelogram approach with that from Hb adduct levels as part of validation of the described approach; (**a**) AUC-GA per AUC-AA, and (**b**) AUC-DEB per AUC-EB [cf. Motwani and Törnqvist^6^].

As a validation of the parallelogram approach, the predicted AUC of the reactive metabolite per AUC of the precursor compound was compared with the corresponding values calculated from measured Hb adduct levels *in vivo*. Notably, the values of AUC-GA estimated from the parallelogram approach *vs*. that from Hb adduct measurements were within a close range; 18 *vs*. 49 nM·h/μg AA per kg bw in human, and 48 *vs*. 18 nM·h/μg AA per kg bw in rat, respectively (Tables 2 and 3). For comparison between species, as explained above, an AUC ratio of GA-to-AA would give a better estimate; for both human and rat, the ratio of AUC-GA per AUC-AA predicted from the parallelogram approach and that from measured Hb adduct levels was similar, within an arbitrary factor of 3, as shown in Fig. 4a. The ratio between species (rat-to-human) of AUC-GA per AUC-AA from the parallelogram approach and Hb adduct levels was 16 and 2.3, respectively, i.e. the prediction indicated a 7 × larger difference between the species than obtained in the published *in vivo* studies used for comparison (Fig. 4a). This is not a large difference considering the above mentioned uncertainties in the estimation of AUCs from the parallelogram approach.

In our first application of the parallelogram approach, AUC-DEB was predicted in a case study on BD exposure. The AUC-DEB was predicted in the mammalian species human, rat and mouse to 0.078, 1.26 and 41 nM·h, respectively, for 1 ppm·h of BD exposure^6^. The corresponding values of AUC-DEB calculated from measured Hb adducts levels were in good agreement; 0.023, 1.37 and 38 nM·h/ppm·h BD, respectively^6^, derived from measurements in occupationally BD-exposed humans and exposure experiments with rat and mouse to BD^7,33–35^. In the case of BD, the relatively large inter-species differences in metabolism were an advantage for testing of the approach. Further, the ratio for rat-to-human of AUC-DEB per AUC-EB from the parallelogram approach was 2.2, which was similar to the rat-to-human ratio calculated as 4.3 from measured Hb adduct levels (Fig. 4b).

AUC *in vivo* could be measured by different methods, e.g. direct measurements in plasma^36^, which could have been used for comparison. However, in the studied cases, the Hb adduct levels have been available as accurate measurements. Even though AUC of the metabolite predicted from the parallelogram approach was in close range to that estimated from the measured Hb adduct levels, there are limitations to the described inter-species approach. One limitation concerns the consideration of the bioavailability of the metabolite by the equation. Inclusion of the AUC of the precursor of both species in the parallelogram equation implies that the bioavailability of the precursor is considered. However, the distribution and direct excretion of the metabolite is reflected by using the AUC of the metabolite in the other species, which is assumed to have only a minor influence (<5%) on the predicted values [cf. Fennell *et al*.^25,26^]. Although the *in vitro* to the *in vivo* extrapolation is simplified in the parallelogram approach by use of AUCs, the disadvantage in its present form is that it still requires *in vivo* data. The simplification is that the approach does not require the *in vivo* descriptors associated with pharmacokinetics, such as blood flow rate, body and liver weight, etc., and chemical parameters related to the compound. Further, this approach, like other pharmacokinetic models for estimation of metabolism, suffers from the uncertainties related to age and diet dependent changes in physiological parameters.

## Discussion

Using animal cancer test for risk estimation requires both inter-species extrapolation of metabolism to genotoxic intermediate and extrapolation from high exposure doses in animals to low exposure doses in humans. It is important to know the relationship between internal dose of the genotoxic metabolite and exposure dose of the precursor compound to be able to use *in vivo* data on genotoxic/carcinogenic potency for the estimation of risk from exposure doses^37,38^. There are default assumptions for direct extrapolation of inter-species differences in metabolism to be used for cancer risk assessment^39^. A step for improvement is to measure the AUC of a reactive metabolite *in vivo* in blood in the animal species. This could be done by repeated direct measurements in plasma, or more accurately by measurement of stable Hb adducts at a single occasion in exposed rodents, both in cancer studies and metabolism studies^9,40^.

The ultimate cancer-risk increasing agents of carcinogens such as AA and BD, having one or more C–C double bond(s), are their respective genotoxic epoxy metabolite(s) (three epoxy metabolites formed in the case of BD). An accurate estimation of AUC of a compound/metabolite by measuring Hb adducts requires that at least 3 animals are exposed to the (precursor) carcinogen at different exposure doses per species (rats and mice) per sex^9,40^; which as in the case of BD resulted in nearly 100 exposed rodents. Internal doses, for instance of metabolites from AA and BD, could also be estimated by classical physiologically-based pharmacokinetic (PBPK) models^41–43^. Using a comparatively consistent set of parameter values and incorporating available data on Hb adduct levels of AA and GA from known exposure doses of AA, the PBPK model has been developed to predict blood levels of AA and GA in humans and rats^44^. Even though the PBPK modelling resulted in comparable values of AUC of AA and of GA in the different species^27,44^, these approaches involving mathematical modelling are considered to be much more complex and prone to uncertainties from the selected model parameters, data inclusion/exclusion criteria and individual variability^45,46^.

The unique, and simple, parallelogram based approach (Fig. 1, Eq. 1) proposed by Motwani and Törnqvist (2014) has been tested and validated in the present work for estimation of AUC-GA from AA exposure, and in our earlier work for estimation of AUC-DEB from BD exposure^6^. The AUC of the reactive metabolites (GA or DEB) was estimated in human with relatively large certainty from the prediction based on the parallelogram approach in both cases. The validation study showed that the ratio between AUCs of reactive metabolite and precursor predicted was in good agreement with the corresponding AUC ratio of metabolite-to-precursor calculated from measured Hb adduct levels (Fig. 4). For prediction of the AUC of a reactive metabolite, e.g. in human, in the present form the approach requires the following data. From human and rat and/or mice: (i) enzyme kinetic data on formation and elimination of the metabolite that can be obtained *in vitro*, (ii) blood protein adduct levels of the precursor compound; and from the rodent: (iii) blood protein adduct levels of the metabolite. Accuracy in the measurements for estimation of enzyme kinetics *in vitro* and protein adduct levels *in vivo* influences the accuracy of AUC value predicted from the parallelogram approach.

The cobalamin-adduct method and modified Edman method are state-of-the art methods for measurements of the epoxide-metabolites and protein adducts, respectively, resulting in relatively high accuracy in the quantification of the studied compounds *in vitro* and *in vivo*. The cobalamin method uses the supernucleophilicity of cob(I)alamin (that reacts ca. 10^5^ × faster than common nucleophiles)^47^ for instantaneous trapping of short-lived and reactive metabolites, resulting in respective alkylcobalamins that can be analyzed by LC-MS/MS^8,20^. This method was applied in the present work for the analysis of GA, as GA-Cbl. For electrophilic compounds/metabolites *in vivo*, measurement of corresponding Hb adducts is a well-established method to derive at respective AUCs in blood. Not many laboratories have this methodology established to perform accurate measurements of blood protein adducts as quantitative biomarkers from low exposure levels in humans^3,7,48^ (and references there in), e.g. from dietary exposure.

The Motwani and Törnqvist equation (i.e. Eq. 1) can be regarded as a new tool within the 3 R framework due to the following advantages: (i) *in vivo* dose of the genotoxic metabolites can be estimated, which is usually difficult to obtain at low exposure levels, as in the case of humans; (ii) the approach is simpler compared to PBPK modelling for the prediction of internal doses; and (iii) the approach has a potential to reduce animal experiments for metabolism studies that are to be used, e.g., in cancer risk assessment. Further, the scope of the approach could be extended so that *in vivo* dose known for one genotoxic compound per exposure unit, for instance in rat, could be extrapolated to *in vivo* dose of another structurally related compound. This would be particularly useful for estimating *in vivo* dose of emerging or newly identified compounds, e.g. in food or as air pollutant, which otherwise might be subjected to traditional animal experiments.

## Methods

### Chemicals and other materials

Acrylamide (AA) was obtained from Merck, Germany. *rac*-Glycidamide (GA) was obtained from Toronto Research Chemicals Inc, Canada. Hydroxocobalamin hydrochloride, cobalt(II) nitrate, sodium borohydride, *rac*-propylene oxide (PO), β-nicotinamide adenine dinucleotide phosphate sodium salt (NADP), glucose-6-phosphate, magnesium sulfate, potassium chloride, and trifluoroacetic acid were obtained from Sigma-Aldrich (Steinheim, Germany). Human and rat liver S9 fraction were purchased from Moltox (Boone, NC, USA); protein content was in the range 10–30 mg/mL, provided by the supplier for every batch. Human S9 was a pooled fraction of five smokers, all male. Rat S9 was from a male SD strain. All experiments concerning liver S9 were performed in accordance to relevant approval at Stockholm University. Stockholm University has obtained a general license from the Swedish Board of Agriculture for the import and use of animal products and by-products for research purposes. No experiments were performed on animals or on humans in this work. Only published *in vivo* data were used which are carefully cited.

### Metabolism experiments

Metabolism experiments were performed using human or rat liver S9 with 7–8 different concentrations in duplicates of AA (20–1500 μM) and GA (2–50 μM) as substrates, under physiological conditions, pH 7.5 at 37 °C, and total volume of 1.5 mL, in accordance with OECD guidelines for testing of chemicals. The metabolic system was similar to that we used in our earlier work^20^ and is described in Supplementary information Note 1. After 15 min incubation with AA as substrate and 10 min with GA as substrate, an aliquot (200 μL) was removed and mixed with ice-cold internal standard PO (3 μM, 200 μL) in methanol that quenched the metabolic activity. The mixture was centrifuged (500 × g, 4 min) and supernatant treated as described below. Control experiments were performed in absence of NADP when studying AA to GA metabolism, and in absence of the respective substrate.

### Formation and analysis of alkylcobalamins

The supernatant (300 μL) obtained following centrifugation in the metabolism experiments was added to a cob(I)alamin solution (200 μL) in water. The reaction was continued under argon atmosphere for 20 min, and subsequently a gentle stream of air was passed to oxidize any reduced cobalamin. Analysis of the solution was performed by liquid chromatography tandem mass spectrometry (LC-MS/MS). The MS was operated using electrospray positive-ionization (ESI^+^) and quantification performed using multiple reaction monitoring (MRM) mode. Method for preparation of cob(I)alamin solution from hydroxocobalamin and analysis of the generated alkylcobalamins, GA-Cbl from GA and PO-Cbl from PO, were similar to our earlier work^8^ and are described under Supplementary information Notes 2–4.

### Data analysis and statistics

The area under MRM peaks from GA-Cbl and PO-Cbl were measured with Analyst software version 1.5 from AB Sciex. Using calibration curve obtained for GA using inactivated S9 as matrix, the concentrations of GA in the metabolism studies were determined as described earlier by us^8^. The metabolic data was used to obtain Lineweaver-Burk plot for graphical representation of studied enzyme kinetics. Apparent values for the respective maximum velocity (V_max_) of the enzymatic conversion, the Michaelis constant (K_m_) and standard error were estimated by nonlinear fitting using the Hill function in Origin version 2015 (OriginLab, Northampton, MA, USA).

### Calculation of *in vivo* dose from Hb adduct levels

The *in vivo* dose or AUC of a compound in blood is the concentration integrated over time (Eq. 2).

2 $${\rm{AUC}}={\int }_{{\rm{t}}}{\rm{C}}({\rm{t}}){\rm{dt}}\,$$

A method to infer the AUC of an electrophilic compound or metabolite from measured Hb adduct levels (*A*) has been developed and established by our research group for quantitative monitoring of exposure in humans or for application in animal studies^1,3^. When Hb adduct formation is measured a short time after a single acute exposure, or after incubation *in vitro*, AUC can be calculated using Eq. 3. This equation is based on *A*, and the second-order reaction rate constant (k_Nu_) for the formation of an adduct to a nucleophilic site in Hb from the concerned electrophile.

3 $${\rm{AUC}}=A/{{\rm{k}}}_{{\rm{Nu}}}$$

For calculating the daily AUC from a longer duration of exposure, the daily adduct level increments (*a*) have to be calculated considering the accumulation and elimination of adducts due to lifetime of the erythrocytes (*t*_*er*_). A steady state adduct level (*a* × *t*_*er*_/2) is reached from exposure for longer time than *t*_*er*_. From continuous or subchronic exposure when the exposure time (*t*) is shorter than *t*_*er*_, the accumulated adduct level is calculated [*a* × *t* × (1 − t/2*t*_*er*_)]^49^.

*In vivo* dose or AUC of AA and of GA in human after intake of AA-rich food was obtained from Vikström *et al*.^23^. The study design included nine nonsmokers (three male and six female; 56–85 kg bw) who consumed food prepared at high temperatures such as French fries and crisp bread for 4 days, and the food intake was monitored by use of food diaries. The mean of the total AA intake was estimated to 3050 μg (range 2500–3500 μg) from the food diary information linked to AA concentration in the food stuffs. Participants donated blood at the start and end of the 4 day period. The blood samples were worked-up according to the N-alkyl Edman method for GC-MS/MS analysis of Hb adducts to N-terminal valine from AA and GA (cf. Fig. 2). The measured adduct levels were used to calculate the daily adduct level increment, *a*, and adjusted for intake per kg bw (exposure dose 11 μg AA/kg bw). This gave the daily adduct level increment per exposure dose of AA and GA as 1.4 and 1.02 pmol/g globin per μg AA/kg bw and day, respectively. Subsequently, the adduct level increments and k_Val_ (second-order rate constant for formation of adducts to N-terminal valine as the nucleophilic site in Hb, obtained *in vitro*) were used to calculate (cf. Eq. 3) the AUC of AA and of GA (in nM·h) per exposure dose (μg per kg bw).

For *in vivo* data on AUC of AA and of GA from AA exposure in rats, a study on short-term oral exposure of rats was used^24^. Eleven F344 rats (male; 150–160 g bw) were given AA in drinking water for 1 week, corresponding to a daily exposure dose of 100–2000 μg AA/kg bw similar to doses given in the cancer tests. After which the animals were weighed and sacrificed. Blood samples were collected and AA- and GA-adducts to N-terminal valine in Hb were measured as described above, and recalculated to the daily increment of the adduct level. Using *in vitro* determined k_Val_ and according to Eq. 3, the AUC of AA and of GA in the exposed rodent were calculated per exposure dose of AA.

### Parallelogram approach for estimation of *in vivo* dose

Considering that a reactive metabolite (RX) is formed by metabolic activation of the corresponding precursor (R), the AUC of RX (AUC-RX) is dependent on the metabolic rate of R to RX (*k′*_R→RX_), elimination rate of RX (*k′*_RXel_) and AUC of R (AUC-R) according to Eq. 4.

4 $$\mathrm{AUC}-\mathrm{RX}\,\propto \,\frac{{k\text{'}}_{{\rm{R}}\to {\rm{RX}}}}{{k\text{'}}_{{\rm{RXel}}}}\times {\rm{AUC}}-{\rm{R}}$$

An inter-species extrapolation strategy from rat to human was used for the estimation of AUC-GA per *in vivo* dose of precursor AA, i.e. AUC-AA. For this the AUC-GA in human was inferred from Eq. 5 representing a parallelogram approach that is derived from Eq. 4. As per the parallelogram equation, the AUC-GA from exposure to AA in human can be predicted using *in vitro* metabolic data in human and rat, together with the AUC calculated from measured Hb adduct levels of AA in human and that of AA and GA in rat.

5 $$\frac{{{\rm{AUC}}}^{{\rm{H}}}-{\rm{GA}}}{{{\rm{AUC}}}^{{\rm{R}}}-{\rm{GA}}}=\frac{\frac{{{\rm{EE}}}^{{\rm{H}}}-{\rm{AA}}\to {\rm{GA}}}{{{\rm{EE}}}^{{\rm{H}}}-{{\rm{GA}}}_{{\rm{el}}}}}{\frac{{{\rm{EE}}}^{{\rm{R}}}-{\rm{AA}}\to {\rm{GA}}}{{{\rm{EE}}}^{{\rm{R}}}-{{\rm{GA}}}_{{\rm{el}}}}}\times \frac{{{\rm{AUC}}}^{{\rm{H}}}-{\rm{AA}}}{{{\rm{AUC}}}^{{\rm{R}}}-{\rm{AA}}}$$

AA → GA and GA_el_ represent metabolic transformation of AA to GA and elimination of GA, respectively, and EE is the enzyme efficiency for the corresponding transformation. Human (H) and rat (R) species are indicated as superscripts. AUC-GA in human was extrapolated by Eq. 5 from AUC-GA in rat using the *in vitro* metabolic EE data and AUC-AA in human and rat. Published data on *in vivo* Hb adduct levels per exposure dose unit in human was obtained from Vikström *et al*.^23^ and in rat from Törnqvist *et al*.^24^. Here we also used the equation to calculate the AUC-GA in rat from human data to cross-validate the AUC data and to obtain a relative inter-species comparison of the AUC-GA between human and rat.

### Data Availability

All data generated and analyzed during this study are included in this published article and it’s Supplementary Information.

## Electronic supplementary material

Supplementary Information
