# Supplementary material for: Parallelogram based approach for in vivo dose estimation of genotoxic metabolites in humans with relevance to reduction of animal experiments
Source: Sci Rep. 2017 Dec 14;7:17560. doi: 10.1038/s41598-017-17692-5 (PMC5730592; doi:10.1038/s41598-017-17692-5)
Supplement: Supplementary file 1 — Supplementary Information [file 41598_2017_17692_MOESM1_ESM.doc]

**Parallelogram based approach for *in vivo* dose estimation of genotoxic metabolites in humans with relevance to reduction of animal experiments**

Hitesh V. Motwani^1^, Cecilia Frostne^1^ and Margareta Törnqvist^1^

^1^Department of Environmental Science and Analytical Chemistry, Stockholm University, SE-10691 Stockholm, Sweden

**Supplementary Information**

Contents

**Note 1.** Preparation of S9 system for metabolism studies

**Note 2.** Formation of cob(I)alamin

**Note 3.** Liquid Chromatography

**Note 4.** Mass Spectrometry (Includes **Figure 1.** A representative LC-MRM chromatogram from the metabolism studies showing the alkylcobalamins, GA-Cbl and PO-Cbl.)

**Note 5.** Published data on AUC of AA and of GA

**Note 1. Preparation of S9 system for metabolism studies**

To human or rat liver S9 fraction (150 μL, 1–5 mg proteins) at 4 °C in a 2 mL vial with a screw-tight cap were added aqueous KCl (0.15 M, 300 μL), MgSO_4_ (80 mM, 150 μL), glucose-6-phosphate (50 mM, 150 μL) and NADP (40 mM, 150 μL), and the volume was made up to 1.5 mL with pH 7.5 phosphate buffer. The mixture was incubated for 5 min at 37 °C prior to the addition of the substrate. Different starting concentrations of the substrate, AA (20 μM to 1500 μM, 8×2) or GA (2 μM to 50 μM, 7×2), were added to the mixture and incubation was continued as described in the article.

**Note 2. Formation of cob(I)alamin**

The method for formation of cob(I)alamin has been described earlier [Motwani and Törnqvist (2011)]. A similar methods was used here where hydroxocobalamin (5 mM in water, 200 μL) and cobalt(II) nitrate (25 mM in water, 20 μL) were added to an amber vial sealed with a septum. The solution was degassed with argon for 10 min. Aqueous sodium borohydride (150 mM, 50 μL) was added, and the mixture was agitated by argon bubbling. After about 2 min, cob(I)alamin formation was judged to be complete.

**Note 3. Liquid Chromatography**

A Shimadzu Prominence LC-system coupled to an API 3200 Q trap triple quadrupole mass spectrometer (Applied Biosystems) was used. The alkylcobalamins, GA-Cbl and PO-Cbl, were analyzed using an ACE 5 C18, 10 mm × 1 mm precolumn that was joined to an automated switching valve connected with an ACE 5 C18, 150 mm × 1 mm analytical column (both columns purchased from Scantec Lab AB, Sweden), which was coupled to the mass spectrometer. The analytes were eluted with gradient conditions using two mobile phase systems referred to as solution A and solution B. Solution A contained 5% acetonitrile in water and solution B contained 70% acetonitrile in water with 0.1% trifluoroacetic acid in each solution. A linear gradient was used from 5% to 30% solution B in 10 min, which was increased to 70% in 7 min, followed by 70% to 100% in 1 min which was held for 5 min before re-equilibrating the column with the initial concentration of the mobile phase. The injection volume was 10 μL with a flow rate of 150 µL/min. To start with, the switching valve was in the loading position for 5 min, which caused the excess hydroxocobalamin and other unwanted waste from the injected sample to pass the precolumn directly to the waste. After 5 min of injection, the valve was switched to the eluting position, and the flow system entered the analytical column, where the two alkylcobalamins were separated.

**Note 4. Mass Spectrometry**

The mass spectrometer was operated using an electrospray ionization source in the positive ion mode (ESI^+^). Acquisition and data processing from the mass spectrometer were performed using the Analyst software, version 1.5, from AB SCIEX. Instrument settings for the ESI were: Collision energy 30 eV, Ion source temperature 400 °C, declustering potential 25 V, entrance potential 7.5 V, collision cell exit potential 25 V, ion spray voltage 5500 V, curtain gas 40, collision gas 5, and ion source gas 15 (latter three are arbitrary values from the Analyst software). Quantitative analysis of processed samples was performed using multiple reaction monitoring (MRM) mode with the following transitions: GA-Cbl m/z 709.8 → 665.5 and PO-Cbl m/z 695.2 → 665.5 (cf. **Fig. 1** below).


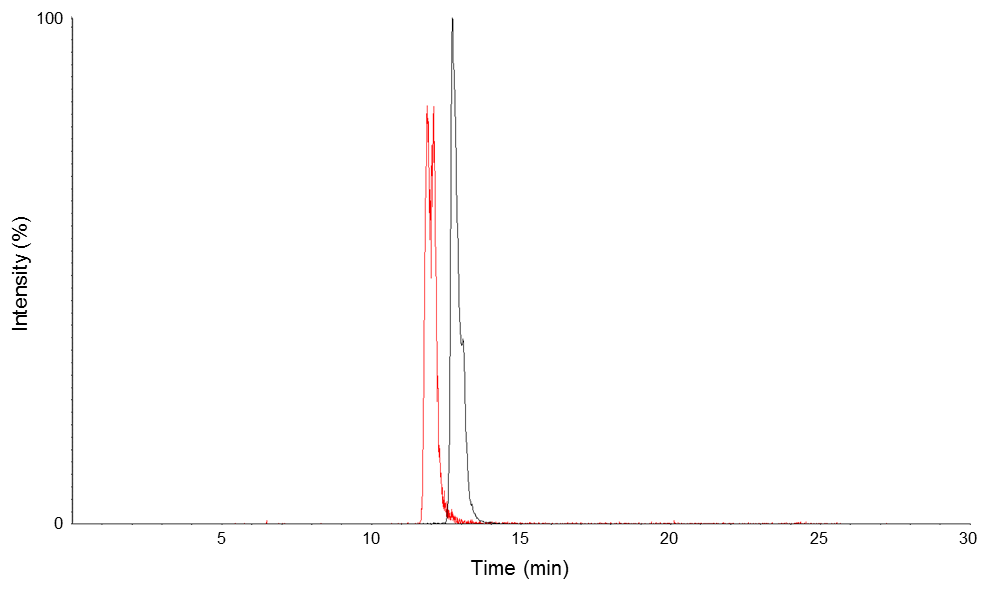


**Figure 1.** A representative LC-MRM chromatogram from the metabolism studies showing the alkylcobalamins, GA-Cbl (left peak) and PO-Cbl (right peak).

**Note 5. Published data on AUC of AA and of GA**

Given below are the values of AUC-GA per AUC-AA calculated from literature data on AUCs of AA and GA normalized to AA dose in humans and male F344 rats. All studies refer to AUCs estimated from Hb adduct measurements of AA or GA, except for one [Doerge et al. (2005)] that was by direct measurements.

|  | AUC-AA nM⋅h/ μg AA per kg bw | AUC-GA nM⋅h/ μg AA per kg bw | AUC-GA per AUC-AA |
| --- | --- | --- | --- |
| **Humans** |  |  |  |
| Vikström et al. (2011)^a^ | 212 | 49 | 0.23 |
| Vikström et al. (2011)^b^ | 120 | 21 | 0.18 |
| Fennell et al. (2005)^c^ | 246 | 60 | 0.25 |
|  |  |  |  |
| **Rats** |  |  |  |
| Törnqvist et al. (2008)^d^ | 34 | 18 | 0.53 |
| Tareke et al. (2006)^e^ | 23 | 14 | 0.63 |
| Doerge at al. (2005)^f^ | 18 | 19 | 1.06 |

^a^Repeated daily dose through food of ca. 11 μg AA/kg bw (n = 9, males and females) during 4 days. Used in the present study for inter-species comparison and validation of the parallelogram approach.

^b^Repeated daily dose through food of ca. 2.5 μg AA/kg bw (n = 9, males and females) during 28 days.

^c^Single dose in drinking water at 500, 1000 and 3000 µg AA/kg bw (n = 6, males).

^d^Repeated daily dose through drinking water at 500 and 2000 μg AA/kg bw (n = 6, males) during one week. Used in the present study for inter-species comparison and validation of the parallelogram approach.

^e^Based on single dose at 0.1 mg AA/kg bw (males) [Tareke et al. (2006) presented data on adduct levels and AUCs as graphs, hence required extraction of values by regression analysis; cf. DeWoskin et al. (2013) for further details].

^f^Single dietary exposure through food of ca. 100 µg AA/kg bw (n = 7, males).
